# Impact of Sampling Variability When Estimating the Explained Common Variance

**DOI:** 10.1177/01466216221084215

**Published:** 2022-04-15

**Authors:** Björn Andersson, Hao Luo

**Affiliations:** 1 205777 University of Oslo, Oslo, Norway; 2 25809 The University of Hong Kong, Hong Kong

**Keywords:** dimensionality, factor analysis, standard errors, psychometrics, scale construction, bifactor models

Assessing multidimensionality of a scale or test is a staple of educational and psychological measurement. One approach to evaluate approximate unidimensionality is to fit a bifactor model where the subfactors are determined by substantive theory and estimate the explained common variance (ECV) of the general factor. The ECV says to what extent the explained variance is dominated by the general factor over the specific factors, and has been used, together with other methods and statistics, to determine if a single factor model is sufficient for analyzing a scale or test (Rodriguez et al., 2016). In addition, the individual item-ECV (I-ECV) has been used to assess approximate unidimensionality of individual items (Carnovale et al., 2021; Stucky et al., 2013). However, the ECV and I-ECV are subject to random estimation error which previous studies have not considered. Not accounting for the error in estimation can lead to conclusions regarding the dimensionality of a scale or item that are inaccurate, especially when an estimate of ECV or I-ECV is compared to a pre-specified cut-off value to evaluate unidimensionality. The objective of the present study is to derive standard errors of the estimators of ECV and I-ECV with linear confirmatory factor analysis (CFA) models to enable the assessment of random estimation error and the computation of confidence intervals for the parameters. We use Monte-Carlo simulation to assess the accuracy of the derived standard errors and evaluate the impact of sampling variability on the estimation of the ECV and I-ECV.

In a bifactor model for J items, denote $X_{j}$ , j = 1, …, J , as the observed variable and let G denote the general factor. We define the S subfactors $F_{s}$ , s∈{1,…, S} , and $J_{s}$ as the set of indicators for each subfactor. Each observed indicator $X_{j}$ is then defined by the multiple factor model (McDonald, 2013)

(1) $$X_{j}=\mu_{j}+\lambda_{Gj}G+\lambda_{sj}F_{s}+\epsilon_{j},$$

where $\lambda_{Gj}$ and $\lambda_{sj}$ are the factor loadings, $\mu_{j}$ denotes the item mean, and $\epsilon_{j}$ is a random error term with variance $\psi_{j}^{2}$ . In a bifactor model, G and each $F_{s}$ are independent of each other and the error terms are independent of each other and with G and each $F_{s}$ .

The explained common variance for CFA models is equal to (Rodriguez et al., 2016)

(2) $$ECV=\frac{\sum_{j=1}^{J}\lambda_{Gj}^{2}}{\sum_{j=1}^{J}\lambda_{Gj}^{2}+\sum_{s=1}^{S}\sum_{j\in J_{s}}\lambda^{2}}.$$

In the literature, it has been suggested that ECV values higher than 0.7 to 0.8 indicate sufficient unidimensionality of the scales to adopt a unidimensional model (Rodriguez et al., 2016). To further evaluate the unidimensionality of a specific item score, another statistic, the explained common variance of an item ( $I-ECV_{j}$ ), was suggested and is defined as (Stucky et al., 2013)

(3) $$I-ECV_{j}=\frac{\lambda_{Gj}^{2}}{\lambda_{Gj}^{2}+\lambda_{sj}^{2}}.$$

The ECV and $I-ECV_{j}$ are functions of the factor loadings and subject to sampling variability when estimating the unknown parameters. However, this has not been considered in prior studies and the impact of random estimation error on the ECV and $I-ECV_{j}$ is unknown. Here, we provide a method for estimating the sampling variability of these statistics based on standard asymptotic theory. Assuming that the estimator $\hat{\theta }$ of the CFA model parameters θ fulfills $\sqrt{n}\left(\hat{\theta }-\theta \right)\overset{D}{\to }\;N\left(0,\;\sum^{\text{​}}\right)$ as the sample size n tends to infinity, we can approximate the variance of the estimators of ECV and $I-ECV_{j}$ by applying the delta method (Ferguson, 1996), such that $\sqrt{n}\left(g\left(\hat{\theta }\right)-g\left(\theta \right)\right)\overset{D}{\to }N\left(0,\frac{\partial g}{\partial \theta }\sum^{\text{​}}{\left(\frac{\partial g}{\partial \theta }\right)}^{\prime }\right)$ where g is either ECV or $I-ECV_{j}$ , continuously differentiable functions of $\hat{\theta }$ . Define λ as the vector of all factor loadings and define $\lambda_{G}$ as the vector of all factor loadings for the general factor. We require the derivatives of the ECV and $I-ECV_{j}$ with respect to the unknown parameters and obtain, for any factor loading λ∈λ

(4) $$\begin{array}{c} \frac{\partial ECV}{\partial \lambda }=\frac{1\left(\lambda \in \lambda_{G}\right)2\lambda \left(\sum_{j=1}^{J}\lambda_{Gj}^{2}+\sum_{s=1}^{S}\sum_{j=1}^{J_{s}}\lambda_{sj}^{2}\right)-\left(\sum_{j=1}^{J}\lambda_{Gj}^{2}\right)2\lambda }{{\left(\sum_{j=1}^{J}\lambda_{Gj}^{2}+\sum_{s=1}^{S}\sum_{j=1}^{J_{s}}\lambda_{sj}^{2}\right)}^{2}}\; \end{array}$$

and

(5) $$\begin{array}{c} \frac{\partial I-ECV_{j}}{\partial \lambda }=\frac{1\left(\lambda =\lambda_{Gj}\right)2\lambda \left(\lambda_{Gj}^{2}+\lambda_{sj}^{2}\right)-\left(\lambda_{Gj}^{2}\right)2\lambda }{{\left(\lambda_{Gj}^{2}+\lambda_{sj}^{2}\right)}^{2}}. \end{array}$$

To assess the accuracy of the standard errors and illustrate the impact of estimation error for ECV and $I-ECV_{j}$ , we simulated data in accordance with a 35-item scale with factor loadings given in Table 3 in Stucky et al. (2013). The bifactor model had one general factor and five independent specific factors that were each measured by 10, 6, 7, 6 and 6 items. All factors were independently generated from standard normal distributions and the residuals were randomly drawn from a normal distribution with mean zero and variances for each item randomly drawn from the U(0.2,0.6) distribution. The true ECV for these data was .615 and the I-ECVs ranged from .171 to .982. We used sample sizes 200, 400, 800 and 1600, and 1000 replications, where the CFA models were estimated using maximum likelihood with the R package lavaan (Rosseel, 2012) and where the R code for computing the ECV and I-ECVs with associated standard errors are available as online Supplementary material.

In Table 1, we present the simulation results for the ECV, showing that the estimators are accurate for all sample sizes. The Monte-Carlo standard errors range from .031 at sample size 200 to 0.011 at sample size 1600 and the asymptotic standard errors based on the delta method with the derivatives from equation (1) are accurate for all sample sizes.

**Table 1.** Monte-Carlo Bias and Standard Errors (MC-SE), Along With Average Asymptotic Standard Errors (SE), for the Estimated Explained Common Variance With Four Sample Sizes.

Sample Size	Bias	MC-SE	SE
200	0.001	0.030	0.029
400	0.001	0.022	0.021
800	−0.000	0.015	0.015
1600	0.000	0.011	0.010

We present the simulation results for the I-ECVs pertaining to the 10 items that measure the first subfactor in Table 2, for sample sizes 200 and 1600. The simulation study included 35 items but we omitted the remaining results since they were highly similar. In Table 2, it can be seen that the sampling variability for I-ECV is generally larger than for the ECV since these statistics are based on the estimated factor loadings of only a single item. Meanwhile, even at sample size 200, the asymptotic standard errors from the delta method using the derivatives from equation (2) are accurate. The results in Table 2 imply that the assessment of item-level unidimensionality with the I-ECV carries with it substantial random error. The largest impact for the first subfactor exists for item 9, where a 95% confidence interval had average length $2\times z_{0.975}\times 0.069\approx 0.270$ with sample size 200, which is quite large for a statistic that ranges between 0 and 1. The average length of a 95% confidence interval for the I-ECV of the same item however reduces to $2\times z_{0.975}\times 0.021\approx 0.082$ with sample size 1600.

**Table 2.** Monte-Carlo Standard Errors With Average Asymptotic Standard Errors in Parentheses, for the Estimated Item-Explained Common Variance of Items 1 to 10 With Sample Sizes 200 and 1600.

Sample Size	Item 1	Item 2	Item 3	Item 4	Item 5	Item 6	Item 7	Item 8	Item 9	Item 10
200	.008	.019	.021	.036	.044	.056	.062	.061	.070	.062
	(.008)	(.018)	(.020)	(.036)	(.045)	(.055)	(.062)	(.061)	(.069)	(.062)
1600	.003	.006	.007	.012	.016	.020	.022	.021	.025	.021
	(.003)	(.006)	(.007)	(.013)	(.016)	(.020)	(.022)	(.022)	(.025)	(.022)

Assessing approximate unidimensionality is commonly done as part of scale development in education and psychology and useful statistics like the ECV help in evaluating unidimensionality. However, this process should be complemented with an assessment of the random error associated with the statistics used. Just like the reporting of reliability coefficients should include standard errors or confidence intervals (Fan & Thompson, 2001), we argue that measures like the ECV and I-ECV should be reported together with an indication of the amount of random error. In this study, we presented a simple solution to assess estimation error for linear factor models and implemented the approach in R for use by interested researchers. Future studies can include the specific results for other statistics commonly used with bifactor models, such as the omega-total and omega-hierarchical coefficients.

## Supplemental Material

sj-pdf-1-apm-10.1177_01466216221084215 - Supplemental Material - Impact of Sampling Variability When Estimating the Explained Common Variance Click here for additional data file. Supplemental Material, sj-pdf-1-apm-10.1177_01466216221084215 for Impact of Sampling Variability When Estimating the Explained Common Variance by Björn Andersson and Hao Luo in Applied Psychological Measurement
